# Supplementary material for: Heterogeneous Multi-Material Flexible Piezoresistive Sensor with High Sensitivity and Wide Measurement Range
Source: Micromachines (Basel). 2023 Mar 23;14(4):716. doi: 10.3390/mi14040716 (PMC10145121; doi:10.3390/mi14040716)
Supplement: Supplementary file 1 [file micromachines-14-00716-s001.zip › micromachines-2285877-supplementary.pdf]

micromachines

MDPI

Article

Heterogeneous Multi-material Flexible Piezoresistive Sensor with High Sensitivity and Wide Measurement Range

Tingting Yu[1], Yebo Tao[2], Yali Wu[3], Dongguang Zhang[3,*], Jiayi Yang[4,*] and Gang Ge[5,*]

[1] School of Aerospace Science and Technology, Xidian University, Xi'an 710071, P. R. China
[2] Intelligent manufacturing College, Jiaxing Vocational & Technical College, Jiaxing 314036, P. R. China
[3] College of Mechanical and Vehicle Engineering, Taiyuan University of Technology, Taiyuan 030024, P. R. China
[4] College of Computer Science and Technology, Xi'an University of Science and Technology, Xi'an 710054, China
[5] Department of Electrical and Computer Engineering, National University of Singapore, Singapore 117583, Singapore
* Correspondence: zhangdongguang@tyut.edu.cn (D. Z.); jyang46@xust.edu.cn (J. Y.); ggeup@nus.edu.sg (G.G.)

Citation: Yu, T.; Tao, Y.; Wu, Y.; Zhang, D.; Yang, J.; Ge, G. Heterogeneous Multi-Material Flexible Piezoresistive Sensor with High Sensitivity and Wide Measurement Range. *Micromachines* **2023**, *14*, 716. https://doi.org/10.3390/mi14040716

Academic Editor: Lin Zhang

Received: 01 March 2023
Revised: 17 March 2023
Accepted: 21 March 2023
Published: 23 March 2023

Copyright: © 2023 by the authors. Submitted for possible open access publication under the terms and conditions of the Creative Commons Attribution (CC BY) license (https://creativecommons.org/licenses/by/4.0/).

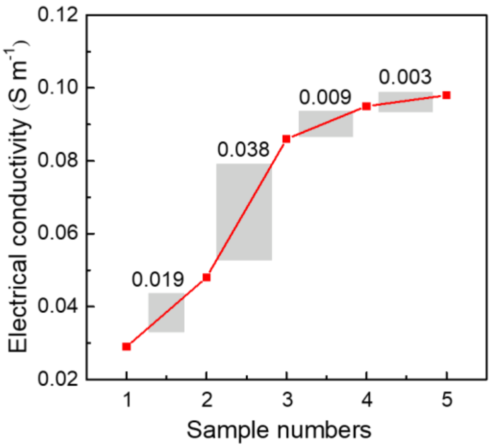

Figure S1. Electrical conductivity of GFs with different dip-coating times.

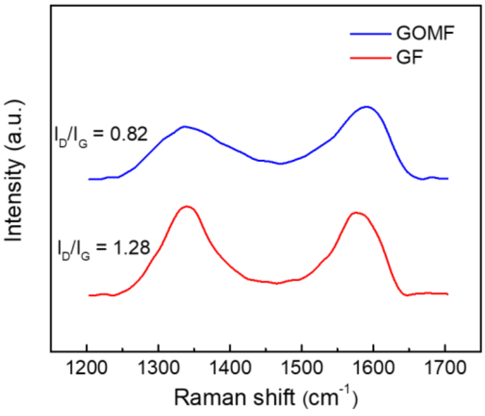

Figure S2. Raman spectrums of the GOMF and the GF.

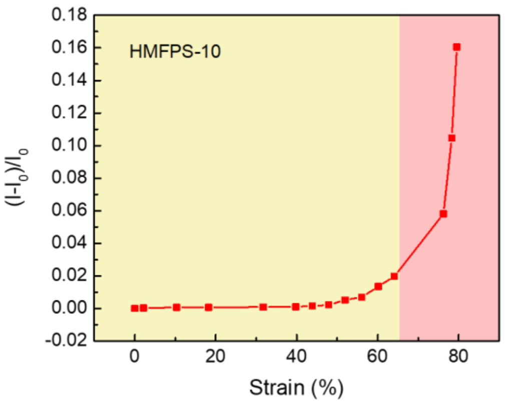

Figure S3. Current responses-strain curves of the HMFPS-10.

Table S1. Comparison between the results of the proposed foam and its counterparts.

Material	Maximum sensitivity (kPa^{-1})	Maximum measurement range (kPa)	Ref.
GF/PDMS	0.695	14122	This work
Magnetic tilt micropillar arraystructured PDMS membrane	0.313	200	[1]
Graphene pressure sensor with random distribution spinosum	25.1	2.6	[2]
Unsymmetrical alveolate PMMA/MWCNTs [1] film	88	10	[3]
Interlocked ordered nanocone array pressure sensor	268.36	0.2	[4]
Carbon nanotube network-coated porous elastomer sponges	0.02	1200	[5]
Hydrophilic hierarchical porous PDMS	0.03	45	[6]
Liquid metal modulated nitrogen-doped graphene nanosheets sponge	476	3.4	[7]
Quasi-hemispherical micropatterned array on the SWCNTs/TPU [2] film	0.02	254.8	[8]
Passive particle jamming variable stiffness material-based flexible capacitive stress sensor	0.023	230	[9]
Porous expandable polyethylene/loofah-like polyurethane sponge material	0.000195	3000	[10]
CNT/SiNPs [3] three-dimensional (3D) printing flexible pressure sensors	0.096	175	[11]
Flexible conductive rGO/TPU foam	0.0152	1940	[12]

[1] PMMA/MWCNTs: polymethyl methacrylate (PMMA)/multiwalled carbon nanotubes (MWCNTs).
[2] SWCNTs/TPU: single-walled carbon nanotubes (SWCNTs)/thermoplastic polyurethane (TPU).
[3] CNT/SiNPs: insulating SiNP carbon nanotubes (CNTs) and fumed silica nanoparticles (SiNPs).
[4] rGO/TPU: reduced graphene oxide (rGO)/thermoplastic polyurethane (TPU) porous foam.

References

[1] Q. Zhou, B. Ji, B. Hu, S. B. Li, Y. Xu, Y. B. Gao, W. J. Wen, J. Zhou, and B. P. Zhou, Tilted magnetic micropillars enabled dual-mode sensor for tactile/touchless perceptions. *Nano Energy* **2020**, 78,

[2] Y. Pang, K. Zhang, Z. Yang, S. Jiang, Z. Ju, Y. Li, X. Wang, D. Wang, M. Jian, Y. Zhang, R. Liang, H. Tian, Y. Yang, and T. L. Ren, Epidermis Microstructure Inspired Graphene Pressure Sensor with Random Distributed Spinosum for High Sensitivity and Large Linearity. *ACS Nano* **2018**, 12, 2346-2354

[3] D. Chen, Z. Liu, Y. Li, D. Sun, X. Liu, J. Pang, H. Liu, and W. Zhou, Unsymmetrical Alveolate PMMA/MWCNT Film as a Piezoresistive E-Skin with Four-Dimensional Resolution and Application for Detecting Motion Direction and Airflow Rate. *ACS Appl. Mater. Interfaces* **2020**, 12, 30896-30904

[4] Y. Lu, Y. He, J. Qiao, X. Niu, X. Li, H. Liu, and L. Liu, Highly Sensitive Interlocked Piezoresistive Sensors Based on Ultrathin Ordered Nanocone Array Films and Their Sensitivity Simulation. *ACS Appl. Mater. Interfaces* **2020**, 12, 55169-55180

[5] S. Kim, M. Amjadi, T. I. Lee, Y. Jeong, D. Kwon, M. S. Kim, K. Kim, T. S. Kim, Y. S. Oh, and I. Park, Wearable, Ultrawide-Range, and Bending-Insensitive Pressure Sensor Based on Carbon Nanotube Network-Coated Porous Elastomer Sponges for Human Interface and Healthcare Devices. *ACS Appl. Mater. Interfaces* **2019**, 11, 23639-23648

[6] C. Parameswaran, R. P. Chaudhary, S. H. Prutvi, and D. Gupta, Rapid One Step Fabrication of Hydrophilic Hierarchical Porous PDMS with Negative Piezopermittivity for Sensing and Energy Storage Applications. *ACS Appl. Polym. Mater.* **2022**, 4, 2047-2056

[7] Y. Li, Y. Cui, M. Zhang, X. Li, R. Li, W. Si, Q. Sun, L. Yu, and C. Huang, Ultrasensitive Pressure Sensor Sponge Using Liquid Metal Modulated Nitrogen-Doped Graphene Nanosheets. *Nano Lett.* **2022**, 22, 2817-2825

[8] Y. J. Zhang, Y. Zhao, W. Zhai, G. Q. Zheng, Y. X. Ji, K. Dai, L. W. Mi, D. B. Zhang, C. T. Liu, and C. Y. Shen, Multifunctional interlocked e-skin based on elastic micropattern array facilely prepared by hot-air-gun. *Chem. Eng. J.* **2021**, 407,

[9] D. G. Zhang, X. X. Wang, Y. L. Wu, H. L. Song, Z. Ma, X. Y. Zhang, X. F. Yang, R. Z. Xing, Y. Li, and J. Y. Yang, Passive Particle Jamming Variable Stiffness Material-Based Flexible Capacitive Stress Sensor with High Sensitivity and Large Measurement Limit. *Adv. Mater. Technol.* **2021**, 6, 7

[10] Z. Zhao, Q. K. Guo, Y. Sun, N. L. An, P. Z. Hui, L. H. Yang, and X. F. Chen, Bioinspired Hierarchical Structure for an Ultrawide-Range Multifunctional Flexible Sensor Using Porous Expandable Polyethylene/Loofah-Like Polyurethane Sponge Material. *Adv. Intell. Syst.* **2023**, 5,

[11] Z. Tang, S. Jia, C. Zhou, and B. Li, 3D Printing of Highly Sensitive and Large-Measurement-Range Flexible Pressure Sensors with a Positive Piezoresistive Effect. *ACS Appl. Mater. Interfaces* **2020**, 12, 28669-28680

[12] X. Z. Lu, T. T. Yu, F. C. Meng, and W. M. Bao, Wide-Range and High-Stability Flexible Conductive Graphene/Thermoplastic Polyurethane Foam for Piezoresistive Sensor Applications. *Adv. Mater. Technol.* **2021**, 6,
